# Mean Arterial Pressure and Risk of Falls Resulting in Hospital Presentation in Older Adults

**DOI:** 10.1093/geroni/igab046.1727

**Published:** 2021-12-17

**Authors:** Sultana Monira Hussain, Michael Ernst, Christopher Reid, Andrew M Tonkin, Johannes Neumann, Thi Phuong Le Thao, Anna Barker, John McNeil

**Affiliations:** 1 Monash University, Melbourne, Victoria, Australia; 2 University of Iowa, Iowa City, Iowa, United States; 3 Curtin University, Perth, Western Australia, Australia; 4 Silver Chain, Melbourne, Victoria, Australia

## Abstract

Utilising data from the ASPirin in Reducing Events in the Elderly trial participants aged 70-years, we estimated MAP and variation in MAP defined as within-individual SD of MAP from baseline and first 2 annual visits. Falls were confined to those involving presentation to a hospital. Cox proportional hazards regression was used to calculate hazard ratio (HR) and 95% confidence interval (CI) for associations with falls. Amongst 16,703 participants (1,540 falls), MAP was not associated with falls irrespective of antihypertensive medication status (all: HR 1.00, 95% CI 0.99-1.01, not on antihypertensive: HR 1.01, 95% CI 0.99, 1.02, on antihypertensive: HR 1.01, 95% CI 0.99-1.02). Amongst 14,818 participants who remained in the study up to year 2 without falls, 1 unit escalation in MAP variability increased the risk (HR 1.01, 95% CI 1.00-1.03). Compared with those in the lowest tercile of variability, those in the middle or highest tercile of variability experienced an increased risk of falling (middle: HR 1.32, 95% CI 1.06-1.65; highest: HR 1.25, 95% CI 1.01-1.55). When stratified for antihypertensive medication status, those receiving diuretics (HR 1.18, 95% CI 1.00-1.39) or beta-blockers (HR 1.37, 95% CI 1.08-1.73) were at increased risk compared to those receiving renin-angiotensin-system acting agents. All results persisted after adjustment for multiple covariates. The association of diuretics and beta-blockers with falls remained significant even after excluding those with history of heart failure. Older community-dwelling adults with high variability in MAP are at increases risk of falls, particularly amongst those receiving beta-blockers or diuretics.

